# Copper tolerance and distribution of epibiotic bacteria associated with giant kelp *Macrocystis pyrifera* in southern California

**DOI:** 10.1007/s10646-015-1460-6

**Published:** 2015-04-18

**Authors:** Julia Busch, Juliana Ribeiro Nascimento, Ana Carolina Rubem Magalhães, Bas E. Dutilh, Elizabeth Dinsdale

**Affiliations:** Biology Department, San Diego State University, San Diego, California USA; Scripps Institution of Oceanography, University of California, San Diego, USA; Marine Biology Institute-CCS, Universidade Federal do Rio de Janeiro, Rio de Janeiro, Brazil; Biology Department, Federal Fluminense University, Niterói, Rio de Janeiro, Brazil; Computer Science Department, San Diego State University, California, USA; Centre for Molecular and Biomolecular Informatics, CMBI, Nijmegen Centre for Molecular Life Sciences, Radboud University Nijmegen Medical Centre, Geert Crooteplein 28, 6526 GA Nijmegen, The Netherlands

**Keywords:** Copper tolerance, Bacterial isolate, Kelp forest

## Abstract

Kelp forests in southern California are important ecosystems that provide habitat and nutrition to a multitude of species. *Macrocystis pyrifera* and other brown algae that dominate kelp forests, produce negatively charged polysaccharides on the cell surface, which have the ability to accumulate transition metals such as copper. Kelp forests near areas with high levels of boating and other industrial activities are exposed to increased amounts of these metals, leading to increased concentrations on the algal surface. The increased concentration of transition metals creates a harsh environment for colonizing microbes altering community structure. The impact of altered bacterial populations in the kelp forest have unknown consequences that could be harmful to the health of the ecosystem. In this study we describe the community of microorganisms associated with *M. pyrifera*, using a culture based approach, and their increasing tolerance to the transition metal, copper, across a gradient of human activity in southern California. The results support the hypothesis that *M. pyrifera* forms a distinct marine microhabitat and selects for species of bacteria that are rarer in the water column, and that copper-resistant isolates are selected for in locations with elevated exposure to transition metals associated with human activity.

## Introduction

Kelp forests are one of the most widespread and productive ecosystems. Found in shallow temperate coastal waters as well as deeper tropical waters, kelp forests are dominated by brown algae from the genus *Laminaria* (Santelices 2007), such as *Macrocystis pyrifera* (giant kelp) and their high productivity provide habitats and nutrients to an entire ecosystem (Egan et al. 2008; Goecke et al. 2010). The nutrient-rich surface of the macroalgae provides a desirable location for epibiotic colonization resulting in higher abundance and diversity of microbial populations (Egan et al. 2008; Blight and Thompson 2008; Armstrong et al. 2001; Lachnit et al. 2011). Kelp produces a matrix of acidic polysaccharides that have a high binding affinity for transition metals such as copper (Davis et al. 2003; Andrade et al. 2004, 2010). While the algal surface is desirable for colonization, in locations where the amount of transition metals is increased by human activity, the bioaccumulation of metals by the kelp may create a stressful environment for bacteria.

Urbanization, industrialization and boating activity can increase the levels of transition metals entering the ocean. In particular, copper-based paint used to prevent marine fouling on boat hulls contributes to elevated levels of transition metals in marinas and bays around the world (Dobalian and Arias 2005; Biggs and D’Anna 2012; Schiff et al. 2006). In 2004 the average copper level in San Diego Bay was 8 ppb (Johnson and Gonzalez 2005), a 36-fold increase of the natural level of 0.225 ppb in ocean water (Blossom 2007). While copper is an essential micronutrient, in high levels it is lethal. The distribution of marine cyanobacteria cells is adversely affected by copper exposure and strains from populated coastal areas are more tolerant to elevated copper levels than those from the open ocean (Stuart et al. 2009; Moffett et al. 2012). However, little research has been conducted on the microbes associated with the kelp forest and the effects of transition metal accumulation. In southern California, the combination of high boating activity and the close proximity of kelp forests to marinas may increase the bioaccumulation of transition metals. To examine whether there was an effect of bioaccumulation of transition metals on microbial populations, we isolated bacteria from the kelp surface and surrounding water at three locations in southern California and described their taxonomic distribution and effect of copper on their growth.

## Materials and methods

**Fig. 1:**
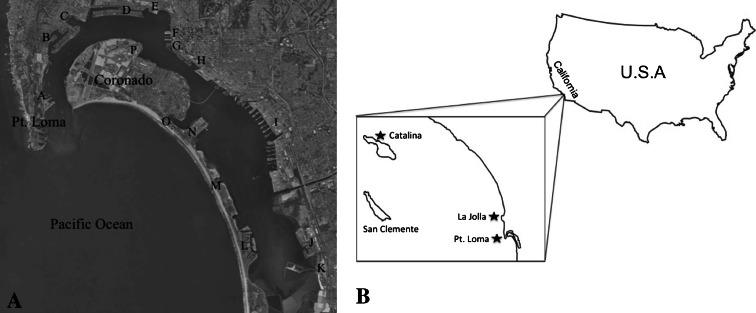
**a** A map of the San Diego Bay labeled with the areas with high boating activity that contribute to the copper load in the water. *a* Navy Submarine Base; *b* Shelter Island; *c* Fisherman’s Landing; *d* Harbor Island; *e* US Coast Guard; *f* B St. Cruise Ship Terminal, Broadway Pier, USS Midway; *g* Tuna Basin; *h* Embarcadero Marina; *i* US Naval Station; *j* Chula Vista Yacht Club; *k* South Bay Power Plant; *l* Coronado Cays; *m* Fiddler’s Cove; *n* Naval Amphibious Base Coronado; *o* Glorietta Bay; *p* Naval Air Station North Island. **b** Sampling locations off the coast of southern California. La Jolla (32°50′3.71″N, 117°16′54.57″W); Pt. Loma (32°39′59.56″N, 117°14′50.62″W); Catalina (33°27′1.89″N, 118°29′12.32″W)

Bacteria were cultured from blades of *M. pyrifera* and surface seawater from three different kelp forest locations in southern California: The Point Loma kelp forest (32°39′59.56″N, 117°14′50.62″W) close to the port of San Diego, La Jolla kelp forest (32°50′3.71″N, 117°16′54.57″W) located adjacent to an urban area but with minimal boating activity, and Santa Catalina Island (33°27′1.89″N, 118°29′12.32″W) located adjacent a marine protected area approximately 70 km from the mainland (Fig. 1). Bacteria associated with the surface of the algae were obtained by washing the blade in 0.02 μm-filtered seawater to remove loosely associated bacteria. After washing, the kelp was wiped across the media plates transferring the viscous surface mucus and associated microbes onto the agar. Bacteria closely associated with the kelp blade tissue were isolated using a technique adapted from Burke et al. (2009) in which kelp surface-associated bacteria were eliminated before plating. The kelp blade pieces were washed in a 1 % bleach solution with gentle agitation for 30 s followed by two washes in 0.02 μm filtered seawater and the blade pieces were applied directly onto the agar. Additional bacterial isolates were obtained by crushing the kelp blade using a sterile mortar and pestle and a 100 μl sample was pipetted onto the media. Bacteria from the water column were obtained from around the kelp blades and 100 μl plated. The media used included TCBS (Neogen Cat. No. 7210A) and Marine Broth (Difco Laboratories Cat. No. 2013-10-31) with 15 g/l agar added. Bacteria were cultured at room temperature and streaked until purification. A total of 600 bacterial isolates were grown and a random set (98) were selected for RFLP analysis and copper tolerance assays.

Bacterial DNA was extracted using nuclei lysis buffer and protein precipitate solution. 16S PCR amplification was performed in which the DNA was denatured for 5 min at 94 °C and amplified through 30 cycles of 94 °C for 1 min, 65 °C for 1 min with 0.5 °C touchdown, 72 °C for 3 min, with a final extension of 72 °C for 10 min. The amplified 16S rDNA was detected by electrophoresis on a 1 % agarose gel with 0.5 μg/ml ethidium bromide in 1× TBE buffer.

To investigate the types of bacteria that were associated with either kelp or the water column at the three locations we conducted a restriction fragment length polymorphism (RFLP) analysis. The 16S sequences of 98 randomly selected bacteria were purified and digested using three restriction endonucleases: ScaI, HaeIII, and MspI. The digested 16S rDNA was run on a 2 % agarose gel followed by staining in ethidium bromide with a final concentration of 0.5 μg/ml. The 16S rDNA PCR product of one isolate from each RFLP group was sequenced using Sanger sequencing. Two sequences were sequenced from Pattern A to investigate whether the restriction pattern were unique to a species. The sequences were aligned with MUSCLE (Edgar 2004) and a maximum likelihood phylogenetic tree was created using RAxML (Stamatakis 2014) with standard settings and 100 bootstrap repetitions. Reference sequences for the phylogenetic analysis where chosen from NCBI after the alignment to identify the type strain of the closes matches to our 16S sequences. The type strains used were of *Alteromonas addita*, *Photobacterium phosphoreum*, *Pseudoalteromonas arctica*, *Pseudoalteromonas citrea*, *Pseudoalteromonas espejiana*, *Psychroserpens burtonensis*, *Ruegeria mobilis*, *Shewanella fidelia*, *Vibrio cyclitrophicus*, *Vibrio mediterranei*, *Vibrio parahaemolyticus*, *Vibrio splendidus*, and *Vibrio wodanis* (accession numbers: AY682202, PHR16SRD4, DQ787199, X82137, X82143, PBU62913, AB255401, AF420312, AM162656, X74710, AF388386, AJ515230, AJ132227 respectively).

The accumulation of copper in kelp was compared for Pt. Loma, near the highest human occupation and Catalina near the lowest level of human occupation in this study. The amount of copper in *M. pyrifera* blades were quantified using an acid digestion method modified from Warnau et al. (1995). The digested samples were analyzed on a Perkin–Elmer DV 4300 inductively coupled plasmid atomic emissions spectrometer (ICP-AES) and conversion calculations were performed to determine the for Cu concentrations. Differences between the copper concentrations at Catalina and Pt. Loma were compared using a student *t* test. Water quality measurements, including temperature, salinity, pH, dissolved oxygen, and chlorophyll-a were taken at surface (~0.5 m) and benthos (~12 m) for both the Pt. Loma and Catalina Island kelp forest collection sites with a MANTA-2 multiprobe (Measurement Specialties, VA, USA). A two sample two sided *t* test for each of the water quality measurements was use to compare the mean from three replicate measurements.

Growth inhibition assays were conducted in order to identify whether the bacteria isolated from different kelp forests had varying tolerance to copper. The median inhibitory concentration (IC_50_) of a bacterial isolate was defined as the lowest concentration of Cu at which growth was half the control value. IC_50_ assays were performed on ten bacterial isolates from Pt. Loma, ten bacterial isolates from La Jolla, and eight bacterial isolates from Catalina. A stock copper solution of CuSO_4_·5H_2_O in deionized water was prepared and sterilized. Bacterial isolates were grown from 1:1000 inoculations of overnight cultures in sterile Zobell Marine Broth 2216 (Difco™) with ranging concentrations of copper. Five replicates of each copper concentration were incubated for 18–20 h at room temperature. Optical density was measured at 550 nm to compare bacterial growth inhibition. To explore the relationship of kelp-associated and seawater isolates and growth inhibition by copper, a one-way ANOVA test was conducted, with the effect of geographic location and sample type (i.e., kelp or seawater) being tested.

## Results

To investigate whether sample type (i.e., kelp tissue or water) or geographic location affected bacterial distribution, a random selection of 98 isolates was classified and 18 different RFLP patterns (labeled A through R) were identified (Table 1). Of the 98 isolates, 57 were *Vibrio* species and were found in both kelp and water from all three locations (Table 1, 2). Bacteria with RFLP Pattern A—most similar to *V. splendidus* were the most abundant and widespread, while Pattern B—most similar to *V. cyclitrophicus* was found across all three locations but not all sample types (Table 1). In addition to geographical differences, eight bacterial species most similar to *Ruegeria* sp., three *Pseudoalteromonas* spp., *Alteromonas* sp., two additional *Vibrio* spp., and *Psychroserpens* sp. were specific to kelp (*n* = 18 isolates), and two groups of bacteria were found exclusively in water (*n* = 7 isolates) including species similar to *V. wodanis* and *P. phosphoreum*.

**Table 1:** Sizes of RFLP fragments obtained from PCR amplified 16S rDNA of marine bacterial isolates

Stock number	Location	ScaI	HaeIII	MspI	Restriction pattern
156	Catalina kelp	1100, 475	310, 290, 210, 190, 150	550, 520, 125	A
157	Catalina kelp	1100, 475	310, 290, 210, 190, 150	550, 520, 125	A
260	Catalina kelp	1100, 475	310, 290, 210, 190, 150	550, 520, 125	A
330	Catalina kelp	1100, 475	310, 290, 210, 190, 150	550, 520, 125	A
131	Catalina water	1100, 475	310, 290, 210, 190, 150	550, 520, 125	A
147	Catalina water	1100, 475	310, 290, 210, 190, 150	550, 520, 125	A
149	Catalina water	1100, 475	310, 290, 210, 190, 150	550, 520, 125	A
189	Catalina water	1100, 475	310, 290, 210, 190, 150	550, 520, 125	A
3	La Jolla kelp	1100, 475	310, 290, 210, 190, 150	550, 520, 125	A
4	La Jolla kelp	1100, 475	310, 290, 210, 190, 150	550, 520, 125	A
5	La Jolla kelp	1100, 475	310, 290, 210, 190, 150	550, 520, 125	A
592	La Jolla kelp	1100, 475	310, 290, 210, 190, 150	550, 520, 125	A
593	La Jolla kelp	1100, 475	310, 290, 210, 190, 150	550, 520, 125	A
594	La Jolla kelp	1100, 475	310, 290, 210, 190, 150	550, 520, 125	A
595	La Jolla kelp	1100, 475	310, 290, 210, 190, 150	550, 520, 125	A
596	La Jolla kelp	1100, 475	310, 290, 210, 190, 150	550, 520, 125	A
598	La Jolla kelp	1100, 475	310, 290, 210, 190, 150	550, 520, 125	A
599	La Jolla kelp	1100, 475	310, 290, 210, 190, 150	550, 520, 125	A
582	La Jolla water	1100, 475	310, 290, 210, 190, 150	550, 520, 125	A
584	La Jolla water	1100, 475	310, 290, 210, 190, 150	550, 520, 125	A
585	La Jolla water	1100, 475	310, 290, 210, 190, 150	550, 520, 125	A
586	La Jolla water	1100, 475	310, 290, 210, 190, 150	550, 520, 125	A
590	La Jolla water	1100, 475	310, 290, 210, 190, 150	550, 520, 125	A
591	La Jolla water	1100, 475	310, 290, 210, 190, 150	550, 520, 125	A
642	La Jolla water	1100, 475	310, 290, 210, 190, 150	550, 520, 125	A
400	Pt. Loma water	1100, 475	310, 290, 210, 190, 150	550, 520, 125	A
**401**	Pt. Loma water	1100, 475	310, 290, 210, 190, 150	550, 520, 125	A
403	Pt. Loma water	1100, 475	310, 290, 210, 190, 150	550, 520, 125	A
404	Pt. Loma water	1100, 475	310, 290, 210, 190, 150	550, 520, 125	A
406	Pt. Loma water	1100, 475	310, 290, 210, 190, 150	550, 520, 125	A
407	Pt. Loma water	1100, 475	310, 290, 210, 190, 150	550, 520, 125	A
252	Catalina water	1100, 475	310, 290, 210, 190, 150	550, 520	B
411	Catalina water	1100, 475	310, 290, 210, 190, 150	550, 520	B
653	La Jolla kelp	1100, 475	310, 290, 210, 190, 150	550, 520	B
643	La Jolla water	1100, 475	310, 290, 210, 190, 150	550, 520	B
**478**	Pt. Loma kelp	1100, 475	310, 290, 210, 190, 150	550, 520	B
479	Pt. Loma kelp	1100, 475	310, 290, 210, 190, 150	550, 520	B
480	Pt. Loma kelp	1100, 475	310, 290, 210, 190, 150	550, 520	B
482	Pt. Loma kelp	1100, 475	310, 290, 210, 190, 150	550, 520	B
501	Pt. Loma kelp	1100, 475	310, 290, 210, 190, 150	550, 520	B
129	Catalina kelp	Uncut	310, 290, 210, 190, 150	550, 520	C
136	Catalina kelp	Uncut	310, 290, 210, 190, 150	550, 520	C
517	Catalina kelp	Uncut	310, 290, 210, 190, 150	550, 520	C
120	Catalina water	Uncut	310, 290, 210, 190, 150	550, 520	C
**121**	Catalina water	Uncut	310, 290, 210, 190, 150	550, 520	C
122	Catalina water	Uncut	310, 290, 210, 190, 150	550, 520	C
137	Catalina water	Uncut	310, 290, 210, 190, 150	550, 520	C
515	Catalina water	Uncut	310, 290, 210, 190, 150	550, 520	C
635	Catalina water	Uncut	310, 290, 210, 190, 150	550, 520	C
522	Pt. Loma kelp	Uncut	500, 290, 190, 125	600, 475, 275, 150	D
523	Pt. Loma kelp	Uncut	500, 290, 190, 125	600, 475, 275, 150	D
524	Pt. Loma kelp	Uncut	500, 290, 190, 125	600, 475, 275, 150	D
525	Pt. Loma kelp	Uncut	500, 290, 190, 125	600, 475, 275, 150	D
526	Pt. Loma kelp	Uncut	500, 290, 190, 125	600, 475, 275, 150	D
527	Pt. Loma kelp	Uncut	500, 290, 190, 125	600, 475, 275, 150	D
**575**	Pt. Loma kelp	Uncut	500, 290, 190, 125	600, 475, 275, 150	D
576	Pt. Loma kelp	Uncut	500, 290, 190, 125	600, 475, 275, 150	D
**583**	La Jolla water	Uncut	500, 290, 190, 125, 100	500, 375, 190, 125	E
644	La Jolla water	Uncut	500, 290, 190, 125, 100	500, 375, 190, 125	E
645	La Jolla water	Uncut	500, 290, 190, 125, 100	500, 375, 190, 125	E
646	La Jolla water	Uncut	500, 290, 190, 125, 100	500, 375, 190, 125	E
483	Pt. Loma kelp	Uncut	500, 290, 190, 125, 100	500, 375, 190, 125	E
484	Pt. Loma kelp	Uncut	500, 290, 190, 125, 100	500, 375, 190, 125	E
485	Pt. Loma kelp	Uncut	500, 290, 190, 125, 100	500, 375, 190, 125	E
521	Pt. Loma kelp	Uncut	500, 290, 190, 125, 100	500, 375, 190, 125	E
**588**	La Jolla kelp	1100, 475	500, 290, 190, 125	550, 520	F
654	La Jolla kelp	1100, 475	500, 290, 190, 125	550, 520	F
657	La Jolla kelp	1100, 475	500, 290, 190, 125	550, 520	F
658	La Jolla kelp	1100, 475	500, 290, 190, 125	550, 520	F
659	La Jolla kelp	1100, 475	500, 290, 190, 125	550, 520	F
581	La Jolla water	1100, 475	500, 290, 190, 125	550, 520	F
132	Catalina water	1100, 475	310, 290, 210, 190, 150	510, 480	G
**187**	Catalina water	1100, 475	310, 290, 210, 190, 150	510, 480	G
253	Catalina water	1100, 475	310, 290, 210, 190, 150	510, 480	G
412	Catalina water	1100, 475	310, 290, 210, 190, 150	510, 480	G
639	La Jolla water	1100, 475	310, 290, 210, 190, 150	510, 480	G
127	Catalina kelp	1100, 475	310, 290, 250, 210, 190, 150	550, 520	H
133	Catalina kelp	1100, 475	310, 290, 250, 210, 190, 150	550, 520	H
134	Catalina kelp	1100, 475	310, 290, 250, 210, 190, 150	550, 520	H
135	Catalina kelp	1100, 475	310, 290, 250, 210, 190, 150	550, 520	H
**641**	La Jolla water	1100, 475	310, 290, 250, 210, 190, 150	550, 520	H
**329**	Catalina kelp	Uncut	310, 290, 210, 190, 150	510, 480, 125	I
416	Catalina kelp	Uncut	310, 290, 210, 190, 150	510, 480, 125	I
139	Catalina water	Uncut	310, 290, 210, 190, 150	510, 480, 125	I
**248**	Catalina kelp	1100, 475	500, 290, 210, 190, 125	510, 375, 180	J
647	La Jolla water	1100, 475	500, 290, 210, 190, 125	510, 375, 180	J
587	La Jolla kelp	850, 475, 225	500, 290, 190, 125	550, 520	K
**589**	La Jolla kelp	850, 475, 225	500, 290, 190, 125	550, 520	K
656	La Jolla kelp	Uncut	310, 290, 190, 150	550, 520	L
**486**	Pt. Loma kelp	Uncut	310, 290, 190, 150	550, 520	L
**142**	Catalina kelp	Uncut	500, 290, 190, 125	510, 375, 180	M
143	Catalina kelp	Uncut	500, 290, 190, 125	510, 375, 180	M
**651**	La Jolla kelp	850, 650	310, 290, 190, 150	510, 480	N
**413**	Catalina kelp	Uncut	310, 290, 210, 175, 150	550, 520	O
**124**	Catalina water	Uncut	390, 310, 290, 190, 150	550, 520	P
**637**	Pt. Loma kelp	Uncut	500, 290, 190, 125, 100	550, 520, 125	Q
**481**	Pt. Loma kelp	Uncut	650, 290,250, 190	800, 450, 125	R

Bold numbers indicate sequenced Isolates from each group

**Table 2:** BLASTn results of 16S rDNA sequences from each RFLP pattern

Sequenced stock number	RFLP pattern	number of isolates	Percent found in kelp	Class	Family	Hypothesized classification	Accession number
ED401	A	31	45.16	Gamma proteobacteria	*Vibrionaceae*	*Vibrio* sp.	KF442438
ED478	B	9	66.6	Gamma proteobacteria	*Vibrionaceae*	*Vibrio* sp.	KF442439
ED121	C	9	33.3	Gamma proteobacteria	*Vibrionaceae*	*Vibrio* sp.	KF442440
ED575	D	8	100	Alpha proteobacteria	*Rhodobacteraceae*	*Ruegeria* sp.	KF442441
ED583	E	8	50	Gamma proteobacteria	*Alteromonadaceae*	*Pseudoalteromonas* sp.	KF442442
ED588	F	6	83.3	Gamma proteobacteria	*Alteromonadaceae*	*Pseudoalteromonas* sp.	KF442443
ED187	G	5	0	Gamma proteobacteria	*Vibrionaceae*	*Photobacterium* sp.	KF442444
ED641	H	5	80	Gamma proteobacteria	*Vibrionaceae*	*Vibrio sp.*	KF442445
ED329	I	3	66.6	Gamma proteobacteria	*Shewanellaceae*	*Shewanella sp.*	KF442446
ED248	J	2	50	Gamma proteobacteria	*Alteromonadaceae*	*Pseudoalteromonas* sp.	KF442447
ED589	K	2	100	Gamma proteobacteria	*Alteromonadaceae*	*Pseudoalteromonas* sp.	KF442448
ED486	L	2	100	Gamma proteobacteria	*Alteromonadaceae*	*Alteromonas* sp.	KF442449
ED142	M	2	100	Gamma proteobacteria	*Alteromonadaceae*	*Pseudoalteromonas* sp.	KF442450
ED651	N	1	100	Gamma proteobacteria	*Vibrionaceae*	*Vibrio* sp.	KF442451
ED413	O	1	100	Gamma proteobacteria	*Vibrionaceae*	*Vibrio* sp.	KF442452
ED124	P	1	0	Gamma proteobacteria	*Vibrionaceae*	*Vibrio* sp.	KF442453
ED637	Q	1	100	Gamma proteobacteria	*Alteromonadaceae*	*Pseudoalteromonas* sp.	KF442454
ED481	R	1	100	Flavobacteria	*Flavobacteriaceae*	*Psychroserpens* sp.	KF442455
Total	18	97		3	5	18	

**Fig. 2:**
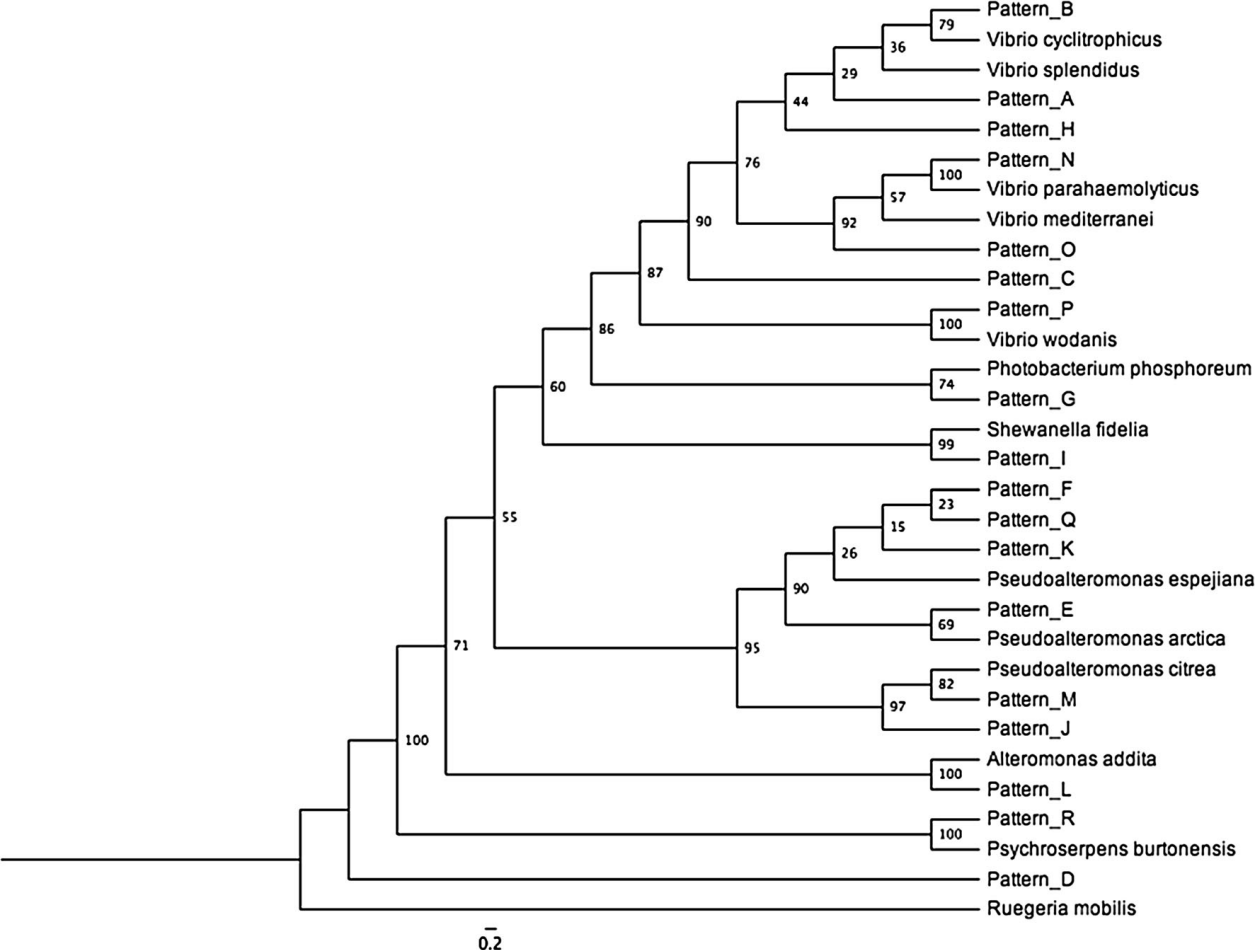
Phylogenetic tree of 16S rDNA sequences created from a MUSCLE alignment using RAxML. Bootstrap values (100 iterations) are indicated at the nodes. The *scale bar* indicates 0.2 mutations per site

Isolates from geographic locations and sample types were distributed evenly throughout the phylogenetic tree (Fig. 2). The *Vibrio* and *Pseudoalteromonas* species clustered into two separated groups and bacteria isolates that had the RLFP Pattern D were not similar to anything in the database and appeared as an outlier in the phylogenetic analysis, suggesting it is a new species based on the 16S rDNA sequence (Fig. 2).

The concentration of copper found in the kelp from Pt. Loma was greater with a mean of 6.21 (± 0.19) ppm compared with the kelp from Catalina which had a mean of 4.33 (±0.08) ppm (***T***_*df*=*4*_ = 10.334, *p* < 0.001). No data was collected from La Jolla. Water quality measurements indicate that the Pt. Loma and Catalina Island kelp forests were similar at surface depths for dissolved oxygen levels (*p* = 0.345) and salinity (*p* = 0.107), and similar at the benthos for chlorophyll-a (*p* = 0.891). In contrast, temperature was higher at the surface in Pt. Loma compared to Catalina [x̄_PtLoma_ = 16.807 (±0.039) °C, x̄_Catalina_ = 16.593 (±0.009) °C] (*p* = 0.027), yet lower in the benthos of Pt. Loma compared to Catalina [x̄_PtLoma_ = 12.210 (±0.015) °C, x̄_Catalina_ = 15.167 (±0.003) °C] (*p* < 0.001). In addition, pH was higher in Catalina at surface [x̄_PtLoma_ = 8.073 (±0.055), x̄_Catalina_ = 8.847 (±0.009)] (*p* = 0.004) and benthos [x̄_PtLoma_ = 8.397 (±0.003), x̄_Catalina_ = 8.783 (±0.007)] (*p* < 0.001). Furthermore, chlorophyll-a was higher at the surface of Pt. Loma [x̄_PtLoma_ = 1.347 (±0.015) mg/l, x̄_Catalina_ = 1.253 (±0.018) mg/l] (*p* = 0.016), while dissolved oxygen was lower [x̄_PtLoma_ = 6.123 (±0.023) mg/l, x̄_Catalina_ = 8.880 (±0.010) mg/L] (*p* < 0.001) and salinity was higher [x̄_PtLoma_ = 45.361 (±0.026) ppt, x̄_Catalina_ = 41.770 (±0.027) ppt] (*p* < 0.001) at the benthos of Pt. Loma compared to Catalina (Table 3).

**Table 3:** Water quality measurements taken with a MANTA-2 multiprobe

	Depth (m)	Temp (°C)	Salinity (ppt)	pH	Chlorophyll-a (mg/l)	Dissolved oxygen (% sat)	Dissolved oxygen (mg/l)
Pt. Loma	0.607 ± 0.047	16.807 ± 0.039	40.308 ± 0.039	8.073 ± 0.055	1.347 ± 0.015	118.067 ± 2.034	9.367 ± 0.153
	13.950 ± 0.183	12.210 ± 0.015	45.361 ± 0.026	8.397 ± 0.003	3.853 ± 0.116	70.267 ± 0.260	6.123 ± 0.023
Catalina	0.230 ± 0.006	16.593 ± 0.009	40.205 ± 0.010	8.847 ± 0.009	1.253 ± 0.018	115.000 ± 0.808	9.177 ± 0.064
	11.490 ± 0.074	15.167 ± 0.003	41.770 ± 0.027	8.783 ± 0.007	3.973 ± 0.766	108.233 ± 0.167	8.880 ± 0.010

Data was logged at surface and benthos depths for each water collection site; Pt. Loma and Catalina Island, California kelp forests. Values displayed are the mean for three replicate measurements, with standard error of the mean (SEM)

**Fig. 3:**
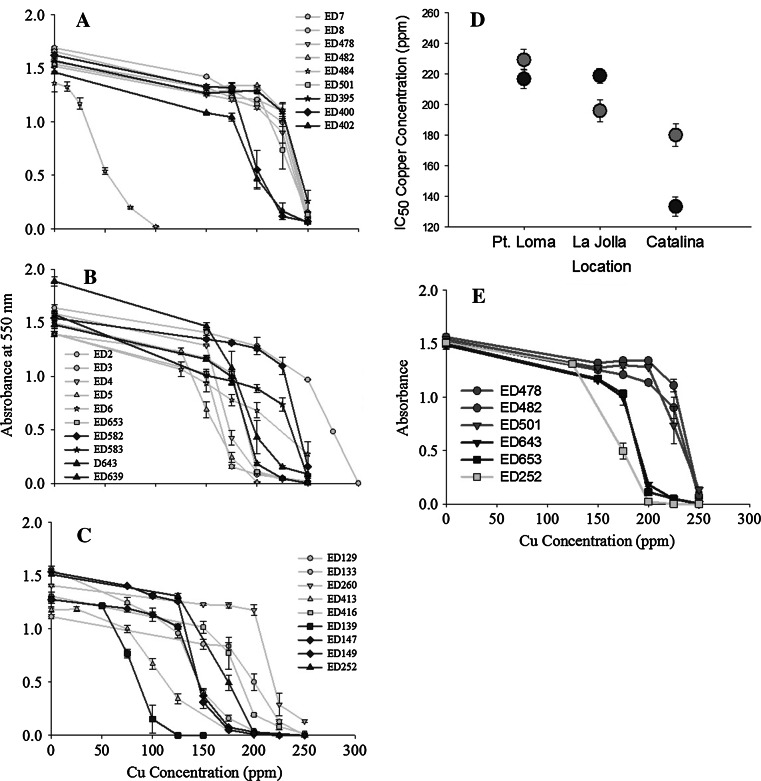
**a** The concentration of copper that inhibited bacterial growth in isolates from Pt. Loma was found to be above 200 ppm in all but one sample. **b** The concentration of copper that inhibited bacterial growth in isolates from La Jolla was found to range from 150 to 275 ppm. **c** The concentration of copper that inhibited bacterial growth in isolates from Catalina was found to range from 100 to 225 ppm. **d** Mean copper inhibitory growth concentrations of *M. pyrifera* (*green*) and seawater (*blue*) associated bacterial isolates across three geographic locations. **e** The copper MIC assay data for bacterial species classified as similar to *V. cyclitrophicus* by 16S rDNA isolated from Pt. Loma (*red*), La Jolla (*blue*), and Catalina (*green*). The isolates from Pt. Loma were the most resistant to copper while the one collected from Catalina was the least copper tolerant. Isolate numbers used in the MIC are from: Pattern A—ED400, ED3, ED4, ED5, ED582, ED147,ED149, Pattern B—D8, ED478, ED482, ED395, ED653, ED643, Pattern C—ED129, Pattern E—ED583, ED484, Pattern G—ED639, Pattern H—ED2, ED133, Pattern I—ED416, ED139, Pattern O—ED413 (Color figure online)

For all kelp forest bacterial isolates, the lowest concentrations of copper in the media resulted in a slight decrease in optical density. After the decrease, bacterial growth was maintained until a steep drop was observed in the graph of absorbance versus copper concentration suggesting the copper concentration had reached an inhibitory level (Fig. 3a–c). The IC_50_ data for Pt. Loma isolates (*n* = 10) showed higher copper tolerance with the majority of growth inhibition occurring between 200 and 250 ppm Cu (Fig. 3a). The bacterial isolates from La Jolla (*n* = 10) showed growth inhibition occurring between 150 and 275 ppm Cu (Fig. 1b). Catalina isolates (*n* = 9) showed growth inhibition occurring between 100 and 225 ppm of Cu (Fig. 3c).

The minimal inhibitory concentration varied with location (F_df=2_ = 41.215 and *p* < 0.001), sample type (F_df=1_ = 4.018 and *p* = 0.047) and there was an interaction between location and sample type (F_df=2_ = 11.394 and *p* < 0.001) (Fig. 3d). The minimal inhibitory concentration was highest at Pt. Loma and the kelp associated bacteria had a higher IC_50_ than the water associated bacteria. The second highest IC_50_ values were found at La Jolla, but the water associated bacteria had higher IC_50_ than the kelp associated bacteria. Catalina showed the lowest IC_50_ and once again the kelp associated bacteria had higher IC_50_ than the water associated bacteria. Trends of the copper tolerance of the community of bacteria were observed in specific bacterial species where five isolates similar to *V. cyclitrophicus* from *M. pyrifera* and seawater showed different values for copper tolerance across the three geographic locations (Fig. 3e). The copper tolerance of isolates was negatively correlated with distance from human urbanization and marinas.

## Discussion

High levels of urbanization, boating and the resultant copper introduced into the water column from antifouling paint may be capable of affecting the structure and function of bacterial populations in kelp forests. Bacterial isolates from Pt. Loma, the location with highest measured copper levels, were able to tolerate copper concentrations significantly higher than bacteria isolated from La Jolla and Catalina, locations with less human activity (Fig. 3d). We showed that the input of transient metals causes a physiological change (tolerance to copper) in the bacteria and the ramifications of these changes on the kelp health will be the goals of future research. Because a range of bacterial species were tested, we show that the development of copper tolerance was location rather than species specific, suggesting that bacteria that lived in a higher copper habitat develop the ability to withstand higher levels of copper.

In addition to the positive correlation between copper pollution and bacterial copper resistance across different species, we observed that strains of a single species, similar to *V. cyclitrophicus*, had higher copper tolerance from Catalina to La Jolla, and the greatest tolerance was found in isolates from Pt. Loma (Fig. 3e). The results of increased copper tolerance in bacteria from areas with higher levels of copper are consistent with findings that *Enterobacteriaceae* strains from marine sediments and seawater have copper resistance directly influenced by pollution (Altug and Balkis 2009). These results suggest that some marine bacteria are able to increase their tolerance to higher levels of copper when living in locations with elevated inputs of transient metals.

Copper in the oceanic water column is reported to be 0. 225 ppb (Blossom 2007) and levels in San Diego Bay was ~8 ppb (Johnson and Gonzalez 2005; Neira et al. 2011), and we found that the copper in the kelp at Pt. Loma and Catalina was high, a mean 6.21 and 4.5 ppm respectively, showing the kelp is concentrating the copper from the water column. The amount of copper in an organism depends on the diffusion across tissue boundaries and the amount to which it is retained in the organism (Neira et al. 2011). The switch in higher copper tolerance in the microbes isolated from the water column compared to the kelp at La Jolla, suggests that at this location the copper may be returning to solution at a faster rate than at Pt. Loma or Catalina. Although the concentrations of copper at which bacterial growth was inhibited for the majority of isolates were above the levels found in *M. pyrifera* from Pt. Loma and Catalina, our results suggests that the transient metals in the environment are correlated with a phenotypic change in the bacteria. In addition previous studies have shown that the brown macroalgae *Ascophyllum* sp. and *Laminaria japonica* can accumulate 80.06 and 100.03 ppt of Cu respectively (Mehta and Gaur 2005),which is 1000 fold greater than the concentrations shown to inhibit growth of the bacterial isolates (Fig. 3). If the antifoulant paint used on boats in the San Diego Bay and other marinas worldwide continue to contribute copper to the water, in addition to other anthropogenic inputs, the algae in adjacent kelp forests such as Pt. Loma are capable of accumulating lethal concentrations of copper for even the most resistant bacterial isolate and these changes in epibiosis may affect kelp health.

A distinct bacterial communities associated with giant kelp were identified. Of the 18 RFLP patterns observed, ten were found in a particular location and ten are specific to either kelp or seawater. These results support the hypothesis that kelp forms a distinct microhabitat that selects for certain species of bacteria that are rare in the surrounding water and we are currently investigating the genotypes of these species to identify why they a specific to either kelp or water column. *Vibrio* species were the dominant organism cultured from the kelp and surrounding water (Table 1). These gram negative, motile rod-shaped species are ubiquitous in marine environments and are found in especially high amounts on eukaryotic host organisms, such as coral and algae (Thompson et al. 2004). This is consistent with our findings as bacterial species from the *Vibrio* genus were the most widespread in terms of location as well as the most abundant. *Pseudoalteromonas* is another well-represented marine Gamma proteobacteria and representatives from this genus have been isolated from both brown and green algae as well as seawater (Egan et al. 2008; Sawabe et al. 1998; Staufenberger et al. 2008). *Shewanella* species have previously been isolated from both seawater and marine organisms and also have the ability to digest agar (Ivanova et al. 2001, 2004). While some species were isolated from both sample types across all sampling sites, many were exclusive to geographical location. Catalina and Pt. Loma, the sites with the least and greatest anthropogenic effects, respectively, have more species that appear to be specific to the locations than La Jolla. Our current research is investigating the non-cultured organisms associated with the kelp using next generation DNA sequencing of metagenomes, which will provide a more complete picture of the kelp microbiome. By culturing bacteria we are able to conduct phenotypic analysis, which are not possible with metagenomics. In addition, we are sequencing the genomes of the cultured bacteria to understand the underpinnings of the phenotypic differences in copper tolerance of the bacteria.

The results of this study suggest that marine microbes are subject to selection at the species level across different geographical locations with varying anthropogenic and industrial influences. Furthermore, kelp appears to select for certain species of bacteria, creating a diverse and distinct microbiome on the algal surface. Kelp forests near industrial areas appear to be decreasing in cover and to date, have failed to recover to pre-1950 levels (Coleman et al. 2008; Connell et al. 2008; Foster and Schiel 2010). The consequences of an altered microbial community could have dramatic effects on the health of the ecosystem and warrants further investigation.
